# Abacavir/Dolutegravir/Lamivudine (Triumeq)–Induced Liver Toxicity in a Human Immunodeficiency Virus–Infected Patient

**DOI:** 10.1093/ofid/ofx122

**Published:** 2017-06-12

**Authors:** Erin S. Christensen, Rupali Jain, Alison C. Roxby

**Affiliations:** 1 Department of Pharmacy Services, and; 2 Division of Allergy and Infectious Diseases, Department of Medicine, University of Washington, Seattle

**Keywords:** abacavir, dolutegravir, hepatotoxicity, human immunodeficiency virus, liver injury, Triumeq

## Abstract

Drug-induced liver injury related to Triumeq (abacavir/lamivudine/dolutegravir) has not been reported in clinical trials. We report a case of hepatotoxicity related to Triumeq exposure in a human immunodeficiency virus–infected patient. Clinicians should remain aware of the risk for acute and late-onset hepatitis with these agents. Close monitoring is recommended.

Hepatotoxicity, characterized by severe elevations in liver transaminases, was frequently observed in older antiretroviral (ART) medications. Nucleoside reverse-transcriptase inhibitors (NRTIs) were historically associated with moderate to severe hepatotoxicity through mitochondrial toxicity, but newer NRTIs, such as abacavir and lamivudine, are associated with a lower incidence of liver injury [1]. This safety concern is often not seen with newer antiretroviral agents such as integrase inhibitors. Several mechanisms of ART-associated hepatotoxicity exist, including hypersensitivity reactions, mitochondrial toxicity, steatosis, direct liver cell stress, and immune reconstitution [2].

We report a case of a 47-year-old Latino male patient with human immunodeficiency virus (HIV) who experienced late-onset hepatotoxicity after starting Triumeq (abacavir/lamivudine/dolutegravir). Eight months after starting Triumeq, routine laboratory testing revealed aspartate aminotransferase (AST) of 180 U/L, alanine aminotransferase (ALT) of 343 U/L, and bilirubin of 0.8 mg/dL. The patient was asymptomatic at this time. Over the next 6 weeks, the AST, ALT, and bilirubin continued to increase, with peaks of 752 U/L, 1004 U/L, and 2.1 mg/dL, respectively. The patient became symptomatic with anorexia secondary to nausea and vomiting, dark urine, fatigue, clay-colored stools, and abdominal pain. Triumeq was discontinued, and the patient’s transaminases and bilirubin immediately started to decrease, normalizing after 8 weeks of no ART.

The patient was treated with Atripla (tenofovir disoproxil fumarate/emtricitabine/efavirenz) for 8 years before switching to Triumeq due to concerns that Atripla was aggravating sleep disturbances and depression. The patient was human leukocyte antigen (HLA) B*5701 negative and had no history of liver disease. He had serologic evidence of prior resolved infection with hepatitis B. Before beginning Triumeq, the patient’s bilirubin was 0.3 mg/dL and AST and ALT were 24 U/L and 23 U/L, respectively, and had been normal for the past 10 years.

The patient denied any recent travel, new exposure to pesticides or toxins, illicit drug use, alcohol intake, or acetaminophen exposure. A toxicology screen was not done. His usual medications included metoprolol, sertraline, cetirizine, and levothyroxine. The patient reported no new partners or risky sexual encounters. He was immune to hepatitis A and had no evidence of hepatitis B reactivation by DNA polymerase chain reaction. He was negative for both hepatitis C virus and hepatitis E virus. His autoimmune work-up was negative, and he had no indication of hemochromatosis. The only significant finding was a low thyroid stimulating hormone (TSH) level of 0.071 uU/mL, but his liver enzymes did not improve once the TSH was normalized. Sertraline was discontinued with no change in transaminases. Serum bicarbonate levels were high throughout, and the anion gap was normal, making lactic acidosis unlikely. He had no symptoms of myopathy. A liver biopsy was performed after the patient developed symptoms, and the biopsy showed hepatic parenchyma with centrilobular hepatocyte dropout and inflammatory infiltrates. No viruses were detected. These findings were consistent with a drug injury. Additionally, based on a validated adverse drug reaction probability scale with scores of −2 to 12, the patient scored a 5, which would be categorized as a “probable” adverse drug reaction [3].

Despite the long interval since Triumeq initiation and a presentation inconsistent with previous reports of abacavir-induced liver toxicity, it was decided to discontinue Triumeq. The patient remained off all ART for 8 weeks. He then started therapy with Genvoya (elvitegravir/cobicistat/emtricitabine/tenofovir alafenamide). On Genvoya, the patient’s AST and ALT remained normal at 23 U/L and 20 U/L, respectively, and his bilirubin was 0.5 mg/dL. Liver tests remained normal 5 months after the incident (AST of 19 U/L, ALT of 16 U/L, bilirubin of 0.4 mg/dL). These findings were reported to the US Food and Drug Administration through a MedWatch report.

Abacavir-induced hepatotoxicity is rare and occurs almost exclusively in HLA B*5701–positive patients [4]. The serum enzyme pattern can be either hepatocellular or cholestatic, and clinically apparent hepatotoxicity is usually anicteric. Additionally, most cases of liver injury associated with abacavir are mild and resolve rapidly within 4 weeks of discontinuation [5]. To our knowledge, only 4 case reports of abacavir-induced liver toxicity in HIV-infected patients with no history of liver problems or alcohol abuse have been reported [6–8]. All reported cases have occurred in patients on a stable nevirapine-based regimen after switching from another nucleoside analog to abacavir.

To our knowledge, there are no case reports published regarding dolutegravir-induced liver toxicity. In clinical trials of dolutegravir, incidence of transaminase elevation was rare. In a phase III trial, <1% of patients experienced transaminase elevations >10 times the upper limit of normal, and only 1 case was thought to be due to a drug-induced liver injury (DILI) [9]. Dolutegravir-induced liver toxicity occurs more frequently in patients coinfected with hepatitis B virus and/or hepatitis C virus [10]. Transaminase elevation in ART-naive HIV-infected patients without viral hepatitis occurred in 3% of patients. Elevations in bilirubin occurred in 12% of patients.

This is the first case report of DILI in an HLA B*5701–negative HIV-infected patient taking abacavir, lamivudine, and dolutegravir. All other case reports of an isolated abacavir-induced liver toxicity have been in patients concurrently taking nevirapine, and we found no reports of dolutegravir-induced liver toxicity. Lamivudine-induced toxicity has been reported but is rare [1]. Although the agent responsible for the DILI cannot be confirmed, it is important to be aware of this rare event with abacavir and/or dolutegravir. The use of combination abacavir-dolutegravir will continue to increase because Triumeq is a single-tablet regimen and first-line treatment option. Clinicians should be aware of the risk for acute and late-onset hepatitis with these agents.
